# Colonic Hemostatic Clip Causing Perforated Acute Appendicitis

**DOI:** 10.5334/jbsr.2417

**Published:** 2021-05-20

**Authors:** Margot Stagnetto, Bruno Coulier, Frederic Pierard

**Affiliations:** 1 Clinique St Luc, 5004 Bouge (Namur), BE; 2 Clinique Saint-Luc, Bouge, BE

**Keywords:** appendicitis, abdomen CT, clip, foreign body, perforation

## Abstract

**Teaching Point:** The advantages of CT over other imaging techniques for the diagnosis of acute appendicitis in adults include the ability to identify foreign bodies that may be involved.

## Case Report

**Figure 1:**
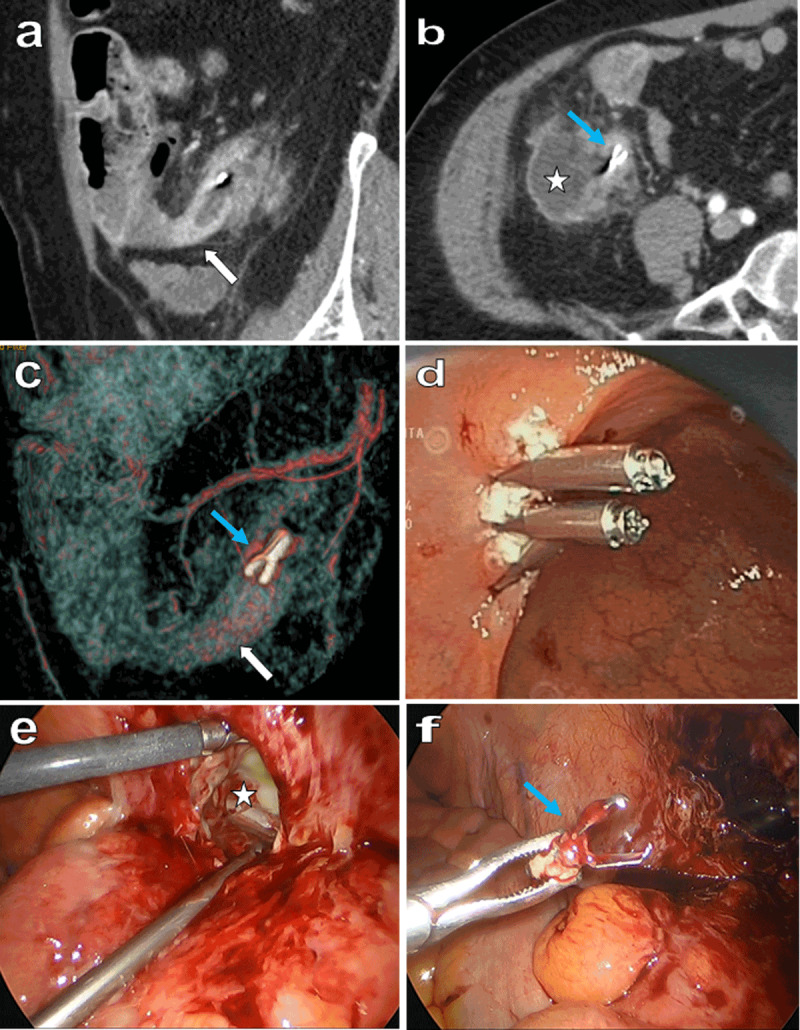


A 65-year-old woman was admitted with pain in the right iliac fossa for three days. Laboratory tests showed elevated C-Reactive Protein (106 mg/L; normal value range <5 mg/L) and white blood cell count (11.400/mm^3^; normal value range 410.000/mm^3^). Contrast-enhanced computed tomography (CT) with sagittal (***Figure 1a***), axial (***Figure 1b***) and volume rendering (VR) (***Figure 1c***) views revealed perforated retrocecal appendicitis (white arrow) with an abscess (***Figure 1b***, white star). An intraluminal metallic foreign body with the typical shape of a hemostatic clip was found (***Figure 1c***, blue arrow). Review of the medical data revealed that the patient had undergone a colonoscopy six weeks previously with a somewhat hemorrhagic resection of a polyp of the cecum requiring two hemostatic clips (***Figure 1d***). One of these had migrated in the appendix causing its perforation. Surgical dissection of the parietal abscess (***Figure 1e***, white star) found the clip (***Figure 1f***) and was completed by radical appendicectomy.

## Comment

Acute appendicitis has an incidence of around 1 per 1000 per year. Rarely it is considered to be related to obstruction or perforation by various foreign bodies including stercoliths, ingested foreign bodies, and rarely, medical devices. Appendicitis caused by the migration of a hemostatic clip as reported here is an extremely rare event, with only one case reported [1].

Foreign bodies tend to settle in the cecum, given its position and low motility. Nevertheless, the incidence of foreign body migration into the appendix is only 0.20.75%. The reason is essentially the small size of the appendix orifice. The anatomical position of the organ is also determinant. So it is unlikely for a foreign body to enter the lumen of a retrocecal appendix as it happened in our patient. Once in the appendix, foreign bodies often cannot be expelled back into the cecum due to insufficient peristaltic motility.

A foreign body can remain in the appendix without stimulating an inflammatory response. Clinical presentation can thus vary from a few hours to several years. In our patient perforating appendicitis developed six weeks after placement of hemostatic clips in the cecum. When the foreign body in relation to appendicitis has a sharp tip, perforation and abscess formation occur in 70% and 31% of cases, respectively.

CT is the best imaging modality for the diagnosis of acute appendicitis in adults (with an average sensitivity and specificity ranging between 72% and 100%) but also to identify foreign bodies.
